# Characterization of cell death induced by vinflunine, the most recent *Vinca* alkaloid in clinical development

**DOI:** 10.1038/sj.bjc.6600025

**Published:** 2002-01-07

**Authors:** A Kruczynski, C Etiévant, D Perrin, N Chansard, A Duflos, B T Hill

**Affiliations:** Division of Experimental Cancer Research, Centre de Recherche Pierre Fabre, 17 avenue Jean Moulin, 81106 Castres, Cedex 06, France; Division of Medicinal Chemistry V, Centre de Recherche Pierre Fabre, 17 avenue Jean Moulin, 81106 Castres, Cedex 06, France

**Keywords:** vinflunine, leukaemia, apoptosis, caspases, Bcl-2

## Abstract

Vinflunine, the most recent *Vinca* alkaloid in clinical development, demonstrated superior antitumour activity to other *Vincas* in preclinical tumour models. This study aimed to define its molecular mechanisms of cell killing in both parental sensitive and vinflunine-resistant P388 leukaemia cells. Vinflunine treatment of these cells resulted in apoptosis characterized by DNA fragmentation and proteolytic cleavage of poly-(ADP-ribose) polymerase. Apoptosis-inducing concentrations of vinflunine caused c-Jun N-terminal kinase 1 stimulation, as well as caspases-3/7 activation. This activation of caspases and the induction of apoptosis could be inhibited by the caspase inhibitor acetyl-Asp-Glu-Val-Asp-aldehyde. Interestingly, the apoptosis signal triggered by vinflunine in these P388 cells was not mediated through Bcl-2 phosphorylation. In addition, when vinflunine resistance was developed in P388 cells, it was associated with resistance to vinflunine-induced apoptosis, as reflected by a loss of capacity to induce DNA fragmentation and PARP degradation, and characterized by increased levels of Bcl-2 and Bfl-1/A1. Therefore, these data indirectly implicate Bcl-2 and Bfl-1/A1 in vinflunine-induced cell death mechanisms.

*British Journal of Cancer* (2002) **86**, 143–150. DOI: 10.1038/sj/bjc/6600025 www.bjcancer.com

© 2002 The Cancer Research Campaign

## 

Vinflunine or 20′, 20′-difluoro-3′,4′-dihydrovinorelbine, is a novel *Vinca* alkaloid selectively fluorinated by superacid chemistry in a rarely exploited region of the velbanamine moiety (Fahy *et al*, 1997). It was selected for clinical development on account of its markedly superior antitumour efficacy *in vivo* in preclinical studies, in a series of experimental models compared to other *Vinca* alkaloids (Kruczynski *et al*, 1998a; Hill *et al*, 1999). Promising results have recently been obtained in Phase I clinical trials, now completed in Europe with three partial responses being identified in patients with advanced breast cancer or renal cell carcinoma (Fumoleau *et al*, 2000). Phase II clinical evaluations in melanoma and renal carcinoma are now on going.

*In vitro* studies have confirmed its mitotic-arresting and tubulin-interacting properties (Kruczynski *et al*, 1998b) and identified differences in tubulin binding relative to the other *Vincas* (Kruczynski *et al*, 1998b; Lobert *et al*, 1998). However, the molecular mechanisms of cell killing by vinflunine remain to be characterized. Anticancer agents are known to trigger apoptotic mechanisms in tumour cells, especially those of haematological origin, although the relative contribution of apoptosis to drug-induced cell death in advanced solid tumours remains controversial (Brown and Wouters, 1999).

Apoptosis, a genetically regulated process triggered by various biological signals, can be transduced through numerous pathways (Wang *et al*, 1999b). However, cells which die by apoptosis exhibit typical morphological changes, DNA fragmentation (Wyllie, 1997) and activate a cascade of aspartate-specific cysteine proteases, namely the caspases. Caspase activity is responsible, either directly or indirectly, for the cleavage of cellular proteins which are characteristically proteolysed during apoptosis (Miller, 1997). For example, once activated, caspase-3 cleaves specific substrates including the nuclear protein poly(ADP-ribose) polymerase (PARP), involved in DNA repair and genome maintenance (Kaufmann *et al*, 1993). Microtubule targeting agents, including taxanes and *Vinca* alkaloids, have been shown to promote apoptosis in cancer cells through a complex process involving many protein kinase signalling pathways (Wang *et al*, 1999b), including activation of c-Jun N-terminal kinases (JNKs) (Stone and Chambers, 2000). Furthermore, a critical role for the Bcl-2 protein family in cell death induced by microtubule damaging agents has been suggested (Wang *et al*, 1999b).

The aim of this study was to determine whether vinflunine-induced cell death in murine P388 leukaemia cells exhibited biochemical characteristics of apoptosis. The contributions of JNK1 signalling, caspases and of Bcl-2 protein family members to vinflunine-induced cell death were also investigated. Furthermore, we examined whether vinflunine resistance developed *in vivo* in P388 cells (P388/VFL) was associated with changes in vinflunine-activated programmed cell death.

## MATERIALS AND METHODS

### Cells

Murine sensitive P388 (National Cancer Institute, Tumour Repository, Frederick, MD, USA) and the *in vivo* established vinflunine-resistant P388/VFL (Etiévant *et al*, 1998) leukaemia cells were collected from the peritoneal cavities of DBA/2 mice (DBA/2JIco, Iffa Credo, L'Arbresle, France), where they were maintained *in vivo*, and adapted to *in vitro* culture conditions in RPMI 1640 medium supplemented with 10% heat-inactivated horse serum, 4 mM L-glutamine, 1.25 μg ml^−1^ fungizone, 100 μg ml^−1^ penicillin-streptomycin, and 20 μM β-mercaptoethanol. The vinflunine-resistant P388/VFL cells established *in vivo* initially were subsequently characterized *in vitro* as showing a 17-fold level of resistance to vinflunine, with marked overexpression of P-glycoprotein (P-gp) associated with reduced accumulation of [^3^H]-vinflunine (Etiévant *et al*, 1998).

### Nuclear staining and apoptotic cell percentage determination

After 24 h of treatment with vinflunine, P388 cells collected by centrifugation were fixed with methanol:acetic acid (3 : 1) for 30 min prior to staining using a Diff-Quik kit (Maurepas, France), based on the Giemsa-May-Grünwald technique, before examination under light microscopy. At least 300 cells were scored for the incidence of apoptosis, in five randomly selected fields and the per cent of apoptotic cells with fragmented nuclei and condensed chromatin relative to the total was calculated.

### DNA fragmentation assay

DNA fragmentation was quantitated according to Bertrand *et al* (1991). Briefly, ^14^C-prelabelled thymidine cells, after exposure to vinflunine, were pelleted and lysed for 30 min at 4°C in 1 ml ice-cold PBS buffer containing 0.5% (v v^−1^) Triton-X-100 and 20 mM EDTA, pH 8. Cellular lysates were centrifuged at 12 000 **g** for 30 min at 4°C to separate low molecular weight DNA fragments (supernatant) from intact chromatin or high molecular weight DNA (pellet). Radioactivity was measured in each collected fraction and the amount of fragmented [^14^C]DNA released into the supernatant was expressed as a percentage of the total. Results are expressed as the drug-specific percentage of DNA fragmented using the formula : (F–F0/100–F0)×100, where F and F0 represent percentages of DNA fragmentation in drug-treated and control cells, respectively.

### Determination of caspase activation in cellular extracts

After *in vitro* vinflunine treatment for 24 h, P388 cells were washed with ice-cold PBS and then lysed for 10 min on ice with 50 mM HEPES, pH 7.4, 0.1% Chaps, 1 mM DTT and 0.1 mM EDTA. Caspase activity in the supernatant (100 μl) was determined using the caspases-3/7 specific colorimetric substrate, acetyl-Asp-Glu-Val-Asp-*p*-nitroaniline (Ac-DEVD-*p*-NA) (Biomol, Plymouth, USA). Briefly, 20 μg protein extract were incubated with 200 μM substrate peptide in 50 mM HEPES, pH 7.4, 100 mM NaCl, 0.1% Chaps, 10 mM DTT, 1 mM EDTA and 10% glycerol. When using the caspases-3/7 inhibitor, Ac-DEVD-CHO (Biomol), cell free extracts were incubated with the inhibitor (200 μM final) for 10 min at 37°C prior to substrate addition. Production of cleaved *p*-nitroaniline from the tetrapeptide substrate Ac-DEVD-*p*-NA was monitored using a Dynatech microplate reader (Guyancourt, France) at 405 nm, allowing quantification of the total DEVD-specific protease activity. Assays were performed in triplicate and results presented as average absorbance±s.e.m.

### Determination of JNK activation in cell extracts

JNK activity was determined by an immunoprecipitation assay (Wang *et al*, 1998). After *in vitro* vinflunine treatment, P388 cells were washed in PBS and lysed for 10 min on ice with 50 mM Tris-HCl, pH 7.5, 150 mM NaCl, 1% NP40, 5 mM NaF, 1 mM sodium orthovanadate. After centrifugation, the cellular extract (supernatant, 1 mg protein) was mixed with 20 μl anti-JNK1 antibody coupled to agarose (Santa Cruz Biotechnology, Santa Cruz, CA, USA) and incubated overnight. The immunocomplex was recovered by sedimentation for 5 min at 20 000 **g**, washed three times with JNK reaction buffer containing 25 mM HEPES, pH 7.5, 25 mM MgCl_2_, 25 mM β-glycerophosphate, and 0.1 mM sodium orthovanadate. The immunoprecipitate was resuspended in 50 μl JNK reaction buffer supplemented with 10 μM ATP, 5 μCi of ^33^P-ATP (2500 Ci mmol^−1^; Amersham, Les Ulis, France) and 0.5 μg GST-c-Jun substrate (amino acids 1–79)-agarose (Santa Cruz Biotechnology) and incubated for 30 min at 30°C. Samples were analyzed by spotting an aliquot of the reaction mixture onto a P81 phosphocellulose membrane (Whatman, Maidstone, UK) which was allowed to dry and then washed three times in 1% (v v^−1^) phosphoric acid in 50% (v v^−1^) ethanol. Radioactivity associated with c-Jun was assessed by scintillation counting. Assays were performed in duplicate, on at least three separate occasions, and results are expressed relative to the activity of controls.

### Western blot analyses

Following *in vitro* treatment with vinflunine, whole cell extracts were subjected to SDS–PAGE before transfer onto nitrocellulose membranes. After blocking non-specific sites, this was probed overnight with an anti-PARP (Serotec, Oxford, UK), an anti-Bcl-2 (Interchim, Montluçon, France), an anti-Bcl-x_L_ (Tebu, Le Perray-en-Yvelines, France), an anti-Bfl-1/A1 (Tebu, Le Perray-en-Yvelines, France), or an anti-Bax antibody (Euromedex, Souffelweyersheim, France), followed by a 1-h incubation with appropriate secondary antibody conjugated to peroxidase (Jackson Immunoresearch Labs, West Grove, PA, USA). When the β-actin loading control was included, probing with an anti-β-actin (Tebu, Le Perray-en-Yvelines, France) or an anti-Bcl-2 family protein antibody was carried out separately. Proteins of interest were visualised by enhanced chemoluminescence (Pierce, Rockford, Il, USA) and quantified using a Bio-Rad (Ivry-sur-Seine, France) MolecularImager.

## RESULTS

### Does vinflunine induce apoptosis in P388 leukaemia parental cells?

#### Vinflunine-induced DNA fragmentation

**Figure 1:**
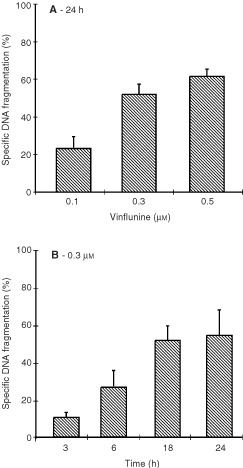
Vinflunine-induced DNA fragmentation in P388 cells. ^14^C-prelabelled thymidine P388 cells were exposed either to 0.1–0.5 μM vinflunine for 24 h (**A**) or to 0.3 μM vinflunine for 3 to 24 h (**B**). Vinflunine-induced fragmented DNA was measured using the intact chromatin precipitation assay, as described in Materials and methods. Results are expressed as means from triplicates, with the associated standard error of the estimate of the mean value (s.e.m.).

Initially, the chromatin precipitation assay was used to assess the cell death response of P388 leukaemia cells to vinflunine treatment. Vinflunine induced fragmentation in P388 cells in a dose- (Figure 1A

) and time- (Figure 1B) dependent manner. DNA fragmentation became apparent following 24 h continuous exposure to 0.1–0.5 μM vinflunine (Figure 1A) and was detectable as early as 3 h with 0.3 μM vinflunine (Figure 1B). Vinflunine-induced DNA fragmentation was also observed using the Tunel assay (data not shown).

#### Vinflunine-activated JNK1

**Figure 2:**
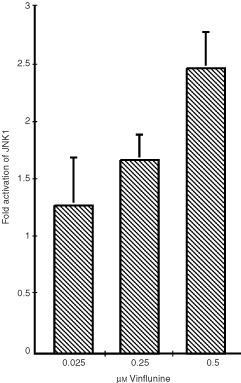
Induction of JNK1 activation by vinflunine in P388 cells. P388 cells were treated with 0.025 to 0.5 μM vinflunine for 6 h. JNK1 activity was determined in cell lysates by immunocomplex assay, as described in Materials and methods. Results, from two independent experiments, are expressed as average increase of JNK1 activity relative to the controls±s.e.m. Statistical evaluation using the Student *t*-test showed that the values for 0.25 and 0.5 μM were significant, with respective *P* values of 0.015 and <0.001.

A 6-h treatment with 0.025, 0.25 or 0.5 μM vinflunine resulted in the activation of JNK1 in these P388 cells in a dose-dependent manner, as illustrated in Figure 2

.

### Vinflunine-stimulated caspase protease activities and caused PARP degradation

**Figure 3:**
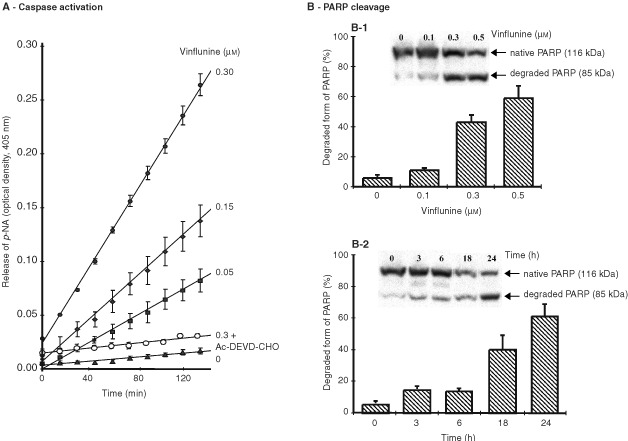
Effects of vinflunine on caspases 3/7 activity and PARP cleavage. (**A**) Enzymatic Ac-DEVD-*p*-NA cleavage activity in P388 leukaemia cell extracts following a 24-h incubation with vinflunine. Twenty μg protein from cell extracts prepared from cells exposed to 0 (▴), 0.05 (▪), 0.15 (⧫), 0.3 (•) μM vinflunine for 24 h were assayed for caspases-3/7 dependent activity using the synthetic substrate Ac-DEVD-*p*-NA. Caspases-3/7 activity was also evaluated in presence of 200 μM Ac-DEVD-CHO, the caspases-3/7 inhibitor (μ), for the cell extracts treated with 0.3 μM vinflunine. Assays were performed in triplicates and results were expressed as average optical density values±s.e.m. (**B**) Cleavage of PARP after either a 24 h-incubation of P388 cells with 0.1–0.5 μM vinflunine (B1) or a 3–24-h incubation with 0.3 μM vinflunine (B2). Vinflunine-induced PARP cleavage was analyzed by Western blot. Results are expressed as means from 2–3 independent experiments of the percentage of degraded form *vs* total (degraded plus native) PARP forms±s.e.m.

Vinflunine, over a concentration range of 0.05–0.3 μM, stimulated caspases-3/7 activities in P388 cells, in a dose-dependent manner (Figure 3A

). The inhibitor, Ac-DEVD-CHO was able to inhibit vinflunine-induced DEVD-specific protease activation, confirming that caspases-3/7 were specifically activated by vinflunine (Figure 3A). Their activation is known to lead to the cleavage of several proteins, with PARP (116-kD) being cleaved to produce an 85-kD fragment during apoptosis (Kaufmann *et al*, 1993). As shown in Figure 3B, PARP and its 85-kD cleaved fragment were detected in P388 cells by immunoblotting, following incubation with vinflunine. A dose-, as well as a time-dependent, cleavage of PARP was observed, with up to 59% PARP cleavage product being recorded after a 24-h exposure to 0.5 μM vinflunine (Figure 3B1), and this was detected as early as 3 h with 0.3 μM vinflunine (Figure 3B2). Overall these results suggest that at least caspases-3/7 play a role in the apoptotic signalling pathway induced by vinflunine in P388 leukaemia cells.

### Inhibition of vinflunine-induced apoptosis by Ac-DEVD-CHO

**Figure 4:**
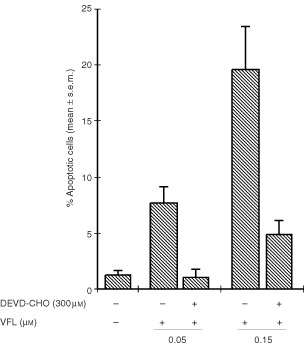
Effects of Ac-DEVD-CHO on vinflunine-induced apoptosis in P388 cells. At 24 h, treated and untreated cells were harvested, and the percentage of apoptotic cells (with condensed and fragmented nucleus) was determined by Giemsa-based staining, as described in Materials and methods. Values are means±s.e.m.

The caspases 3/7 inhibitor Ac-DEVD-CHO inhibited vinflunine-induced apoptosis, as demonstrated by a determination of apoptotic cell number. Optimal inhibition of 86% was noted at a concentration of 300 μM (Figure 4

). These results also provide evidence that at least caspases-3/7 mediate vinflunine-induced apoptosis in P388 cells.

### Effects of vinflunine on Bcl-2 phosphorylation

**Figure 5:**
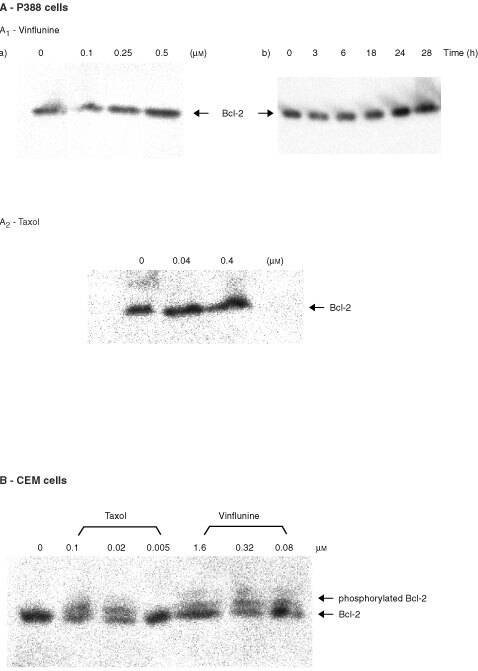
Effects of vinflunine on Bcl-2 phosphorylation. (**A**) Neither vinflunine, nor taxol induced Bcl-2 phosphorylation in P388 leukaemia cells. Western blot analyses of P388 cells after a 24-h exposure to either 0.1–0.5 μM vinflunine (A1) or 0.04–0.4 μM taxol (A_2_), or to 0.3 μM vinflunine for 3–24 h (A_2_). (**B**) Vinflunine and taxol induced Bcl-2 phosphorylation in human CEM leukaemia cells. Western blot analyses of CEM cells after a 24-h exposure to either 0.08–1.6 μM vinflunine or 0.005–0.1 μM taxol.

Neither vinflunine (0.1–0.5 μM) nor taxol (0.04 and 0.4 μM), at equi-cytotoxic concentrations, induced any alteration of the Bcl-2 electrophoretic pattern in P388 cells after a 24-h exposure (Figure 5A

). Taxol was included as a reference since it has been shown to induce Bcl-2 phosphorylation in human leukaemias and solid tumour cells (Haldar *et al*, 1997). Furthermore, a time-course study including treatment of these P388 cells with 0.3 μM vinflunine for 3 h to 48 h also failed to result in any modification of Bcl-2 status. A similar study was then conducted with another cell type, namely human CEM leukaemia cells. After a 24-h exposure to either vinflunine or taxol, immunoblot analyses revealed a dose-dependent modification of the Bcl-2 electrophoretic pattern in these CEM cells, characterized by the appearance of a slower mobility form of Bcl-2 (Figure 5B), similar to that reported after serine-phosphorylation of Bcl-2 (Haldar *et al*, 1995). These findings indicate therefore that microtubule damaging agent-induced Bcl-2 phosphorylation appeared related to the cell type studied.

### Is vinflunine-induced resistance associated with changes in vinflunine-activated programmed cell death or with changes of apoptosis-related protein status?

#### PARP degradation and DNA fragmentation in P388/VFL resistant cells

**Figure 6:**
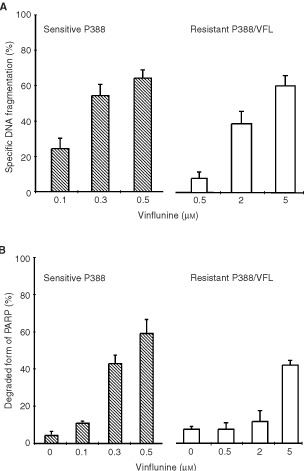
Comparison of vinflunine-induced DNA fragmentation (**A**) and PARP cleavage (**B**) in sensitive P388 and vinflunine-resistant P388/VFL leukaemia cells. See legends to Figure 1 for DNA fragmentation data and to Figure 4 for PARP degradation data.

In order to detect PARP cleavage and DNA fragmentation after a 24-h exposure of P388/VFL resistant cells to vinflunine, it was necessary to increase the vinflunine concentrations 10-fold (i.e., 2–5 μM) relative to those required to induce comparable effects in the sensitive cells (Figure 6A,B

). Therefore, these P388/VFL resistant cells exhibited a 10-fold level of resistance to vinflunine-induced apoptosis, consistent with their 17-fold level of resistance to vinflunine-induced cytotoxicity.

#### Status of Bcl-2, Bcl-x_L_, Bfl-1/A1 and Bax proteins in P388 sensitive and P388/VFL resistant cells

**Figure 7:**
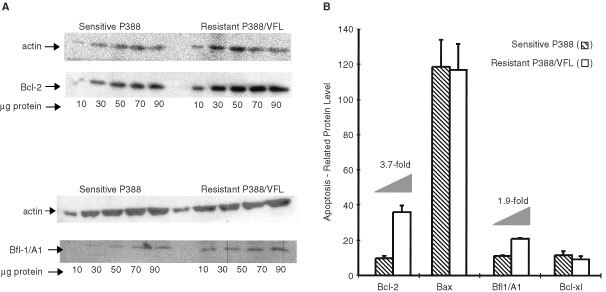
Western blot analyses of the basal levels of Bcl-2 and Bfl-1/A1 in cellular extracts of sensitive P388 or vinflunine-resistant P388/VFL cells (**A**). Quantification of the Western blot analyses of the levels of Bcl-2 , Bax, Bfl1/A1 and Bcl-x_L_, performed using a MolecularImager (**B**).

Western blot analyses were performed to determine whether vinflunine resistance in P388 leukaemia cells was associated with changes in relative endogenous Bcl-2, Bfl-1/A1, Bax, and Bcl-x_L_ expression. As shown in Figure 7

, the basal level of expression of Bcl-2 was 3.7-fold increased in P388/VFL resistant cells relative to the sensitive cells. In addition, a 1.9-fold increase was detected in the basal level of Bfl-1/A1, another anti-apoptotic protein in these resistant cells relative to their sensitive counterparts (Figure 7). The pro-apoptotic protein Bax and the anti-apoptotic protein Bcl-x_L_ however were not differentially expressed in sensitive and resistant cells (Figure 7).

## DISCUSSION

This study aimed to identify the cell killing mechanisms of vinflunine, selected for clinical development on account of its marked *in vivo* antitumour activity and good overall tolerance in preclinical models (Kruczynski *et al*, 1998a; Hill *et al*, 1999) and its quantitatively distinct tubulin interacting properties, compared to other *Vincas* (Kruczynski *et al*, 1998b; Lobert *et al*, 1998).

Our results suggest that vinflunine treatment of P388 leukaemia cells initiates a series of events leading to apoptosis, including DNA fragmentation accompanied by cellular morphological changes specific for apoptosis observed by electron microscopy and formation of apoptotic bodies containing genetic materials identified using a Giemsa-based staining technique (data not shown). Caspase activation and subsequent cleavage of functionally-essential key enzymes also play a central role in the biological processing of apoptosis (Miller, 1997). Caspase-3, required for DNA fragmentation and for some of the typical morphological changes associated with apoptosis (Jänicke *et al*, 1998), cleaves several important cellular targets including PARP (Kaufmann *et al*, 1993). The results presented here show that vinflunine treatment of P388 cells stimulated caspases-3/7 activities and resulted in PARP degradation in a dose- and time-dependent manner. Furthermore, Ac-DEVD-CHO, a caspases 3/7 inhibitor inhibited vinflunine-induced DEVD-specific caspase activity and apoptosis. Therefore, overall these data suggest that vinflunine triggers apoptotic mechanisms involving caspases-3/7 in these P388 leukaemia cells. Involvement of caspases from the caspase-3-like family, as well as PARP cleavage in drug-induced apoptosis has been demonstrated previously for several other microtubule damaging agents, including cryptophycin (Mooberry *et al*, 1997), taxol, vinblastine, vincristine (Srivastava *et al*, 1998) and vinorelbine (Toh *et al*, 1998).

A great deal of evidence implicates JNKs signalling in apoptosis occurring in response to diverse cellular stress stimuli, although it probably has a multifunctional role, varying with the stimulus, the cell type, the duration of enzyme activation, or the specific JNKs isoform(s) involved (Stone and Chambers, 2000). This study reveals that vinflunine induced a dose-dependent JNK1 activation in P388 leukaemia cells. Earlier reports showed that several structurally-distinct anticancer compounds, including vinblastine, doxorubicin and etoposide activated JNKs in human KB-3 carcinoma cells (Osborn and Chambers, 1996). Subsequently confirmation of JNKs activation by vincristine, vinblastine, taxol and colchicine in a variety of cell lines, suggested that this may represent a general stress response to microtubule dysfunction (Wang *et al*, 1998; Stone and Chambers, 2000). Our data showing that JNK1 activation is associated with vinflunine-induced cell death in P388 cells, support these reports.

Mechanisms of drug-induced cell death though appear to be multiple and context-dependent. Thus, vinflunine may trigger other cell death mechanisms under differing experimental conditions. For example, it was suggested (Torres and Horwitz, 1998) that taxol-induced cell killing may result from two mechanisms, involving different mediators depending on the concentration used. A more general review of cell death mechanisms mediated by microtubule damaging agents supports the concept that these are complex processes involving many protein kinase pathways (Wang *et al*, 1999b). The Bcl-2 gene family has been shown to play a crucial role and among multiple genes involved in the regulation of programmed cell death, this gene family stands out for its ability to block apoptosis (Hockenbery *et al*, 1990). Several studies have associated the disorganization of microtubule structure by drugs with mitotic arresting- and apoptosis-inducing activities such as taxol, taxotere, vinblastine, vincristine or the dolastatins with Bcl-2 phosphorylation in various human tumour cell lines (Haldar *et al*, 1997). Bcl-2 can be phosphorylated at serine residues 70 and 87 resulting in loss of its anti-apoptotic function (Basu and Haldar, 1998). Whilst our data revealed no Bcl-2 modifications in these murine P388 leukaemia cells after exposure to either vinflunine or to taxol, similar treatments of human CEM leukaemia cells clearly resulted in Bcl-2 phosphorylation, suggesting that the apoptotic signal triggered by vinflunine in these P388 cells is not mediated via Bcl-2 phosphorylation. Furthermore, the capacity of microtubule-interacting agents to cause Bcl-2 phosphorylation appears to depend more specifically, on its loop domain status. The integrity of both ser-70 and ser-87 residues within the loop domain is necessary for microtubule targeting drug-induced Bcl-2 phosphorylation (Basu and Haldar, 1998). In addition, no phosphorylation of Bcl-x_L_, another anti-apoptotic factor, was detected in P388 cells exposed to vinflunine (data not shown).

Although the relative contribution of apoptosis to drug- and radiation-induced cell killing remains controversial, there are examples in the literature indicating that apoptosis clearly contributes to the overall sensitivity of cells to chemotherapeutic agents (Lowe *et al*, 1994; Rupnow *et al*, 1998), especially for haematological malignancies, where apoptosis appears to be the dominant form of cell death after anticancer drug exposure (Brown and Wouters, 1999). To assess whether apoptosis contributes to the sensitivity of P388 leukaemia cells to vinflunine, PARP cleavage and DNA fragmentation were studied in sensitive P388 and vinflunine-resistant P388/VFL cells concurrently. The concentration of vinflunine required to induce PARP degradation and DNA fragmentation in the P388/VFL cells, with a 17-fold level of resistance to vinflunine, was 10-fold higher than that required to induce the same effects in sensitive cells within the same time frame. This resistance to apoptosis induction may of course be mediated by the drug-efflux protein P-gp, which is overexpressed in these P388/VFL cells and is one mechanism that tumour cells use to escape death induced by chemotherapeutic agents (Germann, 1996). However, our data also identified modifications at the levels of proteins involved in apoptosis regulation, namely the Bcl-2 family members. A higher level of Bcl-2, coupled with an absence of any modification of the expression of the pro-apoptotic factor Bax, was found in vinflunine-resistant P388 cells, associating a high Bcl-2/Bax ratio with vinflunine resistance. These data are consistent with reports of high Bcl-2/Bax ratios being associated with drug resistance, as discussed in a recent review (Fujita and Tsuruo, 2000) although any predictive value of the Bcl-2/Bax ratio in terms of clinical studies remains to be demonstrated (Stoetzer *et al*, 1999). In addition, P388/VFL resistant cells exhibited a higher relative level of Bfl-1/A1 expression, another Bcl-2 family anti-apoptotic member (Zhang *et al*, 2000), which appears to play a role in the apoptotic response of tumour cells to chemotherapy, having been shown to inhibit etoposide-induced apoptosis (Wang *et al*, 1999a). Furthermore, expression of Bfl-1/A1 in NF-kappaB deficient cells provided protection against etoposide and cisplatin treatment (Cheng *et al*, 2000). Our data indirectly implicate Bcl-2 and Bfl-1/A1 in vinflunine-induced cell death mechanisms.

In summary, our data indicate that vinflunine induces apoptosis in murine P388 leukaemia cells, as judged by DNA fragmentation and PARP cleavage. These apoptosis mechanisms involve caspases-3/7 and JNK1 activation, but do not require Bcl-2 phosphorylation. Furthermore, vinflunine resistance in P388/VFL cells is associated with a loss of vinflunine's capacity to induce PARP degradation and DNA fragmentation and with elevated levels of Bcl-2 and Bfl-1/A1. Overall these results complement and extend our previously published data (Etiévant *et al*, 1998; Kruczynski *et al*, 1998a,b; Hill *et al*, 1999), providing an interesting preclinical profile for vinflunine which is currently undergoing phase II clinical testing.
